# Cutaneous Mast Cells Contribute to the Analgesic Effect of Manual Therapy on CCD Rats

**DOI:** 10.1155/prm/2749795

**Published:** 2026-10-26

**Authors:** Jiayang Yu, Pingping Sun, Dong Xie, Xinyu Zeng, Xili Chang, Min Fang, Wuquan Sun, Qingguang Zhu, Chongjie Yao

**Affiliations:** ^1^ Yueyang Hospital of Integrated Traditional Chinese and Western Medicine Shanghai University of Traditional Chinese Medicine Shanghai 200437 China shutcm.edu.cn; ^2^ School of Acupuncture-moxibustion and Tuina Shanghai University of Traditional Chinese Medicine Shanghai 201203 China shutcm.edu.cn; ^3^ School of Rehabilitation Science Shanghai University of Traditional Chinese Medicine Shanghai 201203 China shutcm.edu.cn; ^4^ Engineering Research Center of Traditional Chinese Medicine Intelligent Rehabilitation Ministry of Education Shanghai 201203 China meb.gov.tr; ^5^ Putuo Hospital Shanghai University of Traditional Chinese Medicine Shanghai 200062 China shutcm.edu.cn; ^6^ Shuguang Hospital Shanghai University of Traditional Chinese Medicine Shanghai 201203 China shutcm.edu.cn; ^7^ Research Institute of Tuina Shanghai Academy of Traditional Chinese Medicine Shanghai 200437 China shutcm.edu.cn

**Keywords:** analgesic effect, lumbar disc herniation, manual therapy, mast cells

## Abstract

**Background:**

Lumbar disc herniation (LDH) commonly induces low back and leg pain. Manual therapy (MT) is effective in relieving such pain clinically, but its mechanism remains unclear. Given that acupoint regions are enriched with mast cells that respond to mechanical stimulation, this study explored whether MT alleviates CCD‐evoked neuropathic pain and its association with the modulation of cutaneous mast cells.

**Methods:**

Sprague‐Dawley (SD) rats were randomized into sham, chronic compression of dorsal root ganglion (CCD), MT, and mast cell inhibition (MCI) groups. After CCD modeling, MT was administered at BL57 starting on day 4 for 14 days, while MCI received acupoint cromolyn sodium injection. Paw withdrawal threshold (PWT) was measured. Mast cell activation was assessed using immunofluorescence and toluidine blue staining. Calcitonin gene‐related peptide (CGRP), substance P (SP), Piezo1, and Piezo2 were determined via immunofluorescence; inflammatory cytokines and neurotransmitters by ELISA; expression of MAPK, Piezo1, and Piezo2 by qRT‐PCR. MAPK expressions were also detected by Western blot.

**Results:**

CCD reduced PWT from day 3, whereas MT significantly restored PWT from day 11. In skin, CCD increased mast cell activation, CGRP, SP, tumor necrosis factor (TNF)‐α, interleukin (IL)‐6, IL‐1β, 5‐hydroxytryptamine (5‐HT), and histamine (HA), all of which were attenuated by both interventions. In DRG, CCD elevated p‐ERK, p‐JNK, and p‐p38 MAPK without altering total MAPK expression, and both interventions suppressed MAPK phosphorylation. Piezo2 was upregulated in CCD and reduced by MT and MCI in both tissues. In skin, Piezo1 remained unchanged, while in DRG, CCD downregulated Piezo1 significantly.

**Conclusion:**

MT can relieve CCD‐induced mechanical allodynia, an effect associated with inhibited cutaneous mast cell activation, attenuated peripheral neuroinflammation, and reduced MAPK hyperphosphorylation in the DRG.

## 1. Introduction

Low back and leg pain caused by lumbar disc herniation (LDH) is a common clinical condition, and it is increasingly affecting younger populations in the context of modern lifestyles. The associated chronic pain significantly impairs patients’ quality of life [1]. Manual therapy (MT), also called Tuina in traditional Chinese medicine (TCM), is widely used to treat LDH in clinical practice [2]. Previous studies [3, 4] have demonstrated that deep somatic or visceral pain can manifest as sensitized tender points on the body surface. In TCM, these superficial tender points are identified as acupoints. Accumulating evidence [5, 6] has further revealed neurogenic and peripheral inflammatory alterations at these cutaneous points, supporting an intrinsic correlation between superficial skin changes and somatic pain. Rooted in the TCM theory that stimulating acupoints can treat internal disorders, clinicians routinely deliver MT at such sensitized cutaneous points. Among them, “Chengshan” (BL57) is a classic acupoint for LDH intervention. This point frequently exhibits marked tenderness in LDH patients, and targeted manual manipulation effectively relieves pain symptoms. Our previous clinical studies [7, 8] have also validated the analgesic efficacy of MT at BL57 acupoint in LDH. These findings suggest that mechanical stimulation of sensitized superficial reaction points may contribute to MT‐induced analgesia, but the underlying mechanisms warrant investigation.

Given that acupoint regions are enriched with mast cells and sensory nerve endings, recent studies have indicated that the analgesic mechanism of MT may be associated with the regulation of local neurogenic microenvironment in the acupoint region. It has been reported [9, 10] that LDH‐triggered nerve injury causes sensory terminals to release neuropeptides including substance P (SP) and calcitonin gene‐related peptide (CGRP), thereby activating mast cells distributed in the acupoint region. Upon activation, mast cells release various inflammatory mediators through degranulation, contributing to the development of local neurogenic inflammation and pain sensitization. Therefore, mast cell activation may serve as a key link between peripheral “reaction points” and central pain processing, and its inhibition may be an important pathway through which mechanical stimulation of specific acupoints ameliorates LDH‐related peripheral neuroinflammation. Based on the above pathological cascade, this research hypothesized that MT may alleviate LDH‐induced peripheral sensitization by inhibiting cutaneous mast cell activation and modulating acupoint neuro‐immune regulation. Nonetheless, the underlying mechanism by which MT initiates local biological effects through mechanical stimulation of mast cells remains to be elucidated.

Hence, building on our previous work, this study investigated the effects of MT intervention on pain behavior, mast cell activation, and the expression of local inflammatory cytokines in the skin, MAPK pathway phosphorylation in the dorsal root ganglion (DRG) and the expression of mechanosensitive Piezo1 and Piezo2 channels in the DRG and skin in a rat model of chronic compression of the DRG (CCD). This work aims to investigate whether the analgesic mechanism of MT involves the modulation of cutaneous mast cell–associated neuro‐immune responses, providing a novel peripheral neuro‐immune mechanism for MT‐induced analgesia and providing an experimental foundation for its clinical application.

## 2. Materials and Methods

### 2.1. Experimental Animals

Specific pathogen‐free male Sprague‐Dawley (SD) rats (aged 6 weeks, weighing 220 ± 20 g) were obtained from the Experimental Animal Center of Yueyang Hospital of Integrated Traditional Chinese and Western Medicine, Shanghai University of Traditional Chinese Medicine, and purchased from Shanghai Jihui Experimental Animal Breeding Co., Ltd. (License No. SCXK (Shanghai) 2022‐0009). All experimental procedures strictly followed the protocols approved by the Animal Welfare and Ethics Committee of Shanghai University of Traditional Chinese Medicine (Approval No. PZSHUTCM2505290009). The animal room conditions were regulated to maintain a stable environment, with a temperature of (20 ± 2) °C and relative humidity at 50%–70%. Additionally, a 12‐h light/dark cycle was employed to uphold normal circadian rhythms during the experiment. After 1 week of acclimatization, the experimental procedures began. Rats were randomly divided into four groups: sham, CCD, MT, and mast cell inhibition (MCI) group. While MAPK qRT‐PCR utilized the cohort (*n* = 8) per group, Western blot and Piezo qRT‐PCR were performed on a subset (*n* = 6) per group due to limited yields of high‐quality DRG tissue.

### 2.2. CCD Model

The CCD model was used to simulate the pathological changes induced by LDH [11]. Rats were anesthetized by intraperitoneal injection of 2% sodium pentobarbital (Sigma‐Aldrich, St. Louis, MO, USA) at a dose of 40 mg/kg. After shaving and disinfection with povidone‐iodine, the muscle tissue was bluntly dissected layer by layer along the lumbar back to fully expose the right L4 and L5 intervertebral foramina. Two L‐shaped stainless‐steel rods (4 mm in length, 0.5 mm in diameter) were inserted into the two foramina, respectively. The wound was then sutured layer by layer, and erythromycin ointment was applied to the skin surface to prevent infection. In the sham group, the same surgical procedure was performed except that the rods were not implanted. To confirm successful rod placement, postoperative X‐ray imaging was performed (Samsung XGEO GU60, Seoul, South Korea). Animals exhibiting rod displacement, sensory deficits, or motor dysfunction upon recovery were excluded from the analysis.

### 2.3. Intervention Methods

All interventions began on day 4 post‐CCD surgery, once the model was successfully established and stabilized.

MT group: rats received MT manipulation. The operator placed the right thumb on the junction between the two muscle bellies and the tendon of the right hindlimb gastrocnemius muscle (corresponding to BL57 acupoint) [12] and performed rhythmic pressing and kneading. To standardize the intervention, the operator wore a tactile pressure‐measuring finger sleeve to ensure precise calibration of 5 N applied pressure and 2‐Hz stimulation frequency. The intervention was performed once daily for 10 min, for 14 consecutive days.

Sham and CCD groups: rats received the same grasping and fixation as the MT group but without any manual intervention.

MCI group: rats received acupoint injection. Cromolyn sodium is a well‐established mast cell stabilizer that inhibits degranulation by blocking calcium ion influx through chloride channels on the mast cell membrane [13]. The selected dose (0.02 g dissolved in 1 mL distilled water, 20 µL per day) falls within the established effective range for mast cell stabilization in rats [13, 14]. Intradermal injection at the BL57 acupoint was chosen to achieve local mast cell inhibition specifically at the intervention site, ensuring that the observed effects are attributable to peripheral rather than systemic mast cell modulation. After injection, rats were grasped and fixed on the experimental table for 10 min without other intervention, once daily for 14 days.

### 2.4. Tissue Selection

After the intervention, the right L4 and L5 DRG and skin tissue were collected. A longitudinal incision was made along the posterior midline. The L6 spinous process was identified at the line connecting the bilateral iliac crests, which served as a caudal landmark to locate the L4 intervertebral foramina. After cutting the lumbar vertebrae and opening the spinal canal, the slightly yellowish DRG at the corresponding nerve root exits were carefully removed with fine micro‐forceps. The tissues were rinsed with sterile saline, then separated into two parts: one was snap‐frozen and stored at −80°C for molecular assays, and the other was fixed in 4% paraformaldehyde.

### 2.5. Paw Withdrawal Threshold (PWT) Test

In accordance with previously described and validated methodology, mechanical allodynia was assessed by measuring the PWT of the right hind paw using an electronic von Frey device (IITC Life Science, USA) [15]. The PWT was defined as the force (in grams) that elicited a paw withdrawal response. The probe tip was applied at a constant speed of 0.5 cm/s until a withdrawal response was observed. Behavioral evaluations were performed during the light cycle (09:00–17:00). Prior to testing, the rats were habituated to the experimental environment for 3 days (2 h/day) inside transparent plastic chambers to minimize stress‐related variability. The electronic von Frey filament was then applied vertically to the central plantar surface of the right hind limb through the metal grid floor. A brisk paw withdrawal, licking, or shaking of the stimulated paw was considered a positive response. To avoid sensory sensitization caused by frequent stimulation, an interval of at least 20 s was maintained between two consecutive stimuli. Each rat underwent five replicate trials per testing session. The maximum and minimum values were excluded, and the average of the remaining three values was taken as the session outcome. Baseline measurements were taken once daily for two consecutive days before surgery. Postmodeling measurements were performed on days 3, 7, 11, 14, and 18. All behavioral tests were conducted by experimenters who were blinded to the group allocation.

### 2.6. Immunofluorescence

Immunofluorescence was conducted on skin and DRG tissue sections to assess mast cell activation, neuropeptide expression, and mechanosensitive channel expression. Tissues were fixed in 4% paraformaldehyde for 24 h before paraffin embedding and serial slicing into 4‐μm‐thick sections. Paraffin sections were subsequently deparaffinized and rehydrated before antigen retrieval via microwave treatment in a citric acid buffer (0.01 M, pH 6.0) at 98°C for 15 min, followed by cooling to room temperature for over 60 min. Sections were blocked with 5% bovine serum albumin (BSA) (Beyotime, Shanghai, China) for 30 min at room temperature. They were then incubated overnight at 4°C with primary antibodies against the following targets: mast cell tryptase (1:200, abs148788, Absin, Shanghai, China), CD63 (1:200, abs149061, Absin, Shanghai, China), CGRP (1:600, abs120206, Absin, Shanghai, China), SP (1:200, abs119661, Absin, Shanghai, China), Piezo1 (1:100, 28511‐1‐AP, Proteintech, Wuhan, Hubei, China), and Piezo2 (1:100, 26205‐1‐AP, Proteintech, Wuhan, Hubei, China). Following incubation with the primary antibody, the sections were gradually warmed to 37°C for 45 min. After rinsing, they were incubated with appropriate fluorescence‐conjugated secondary antibodies (1:100, Sangon Biotech, Shanghai, China) for 1 h at room temperature in the dark. Nuclei were counterstained with DAPI (Beyotime, Shanghai, China) for 2 min. Images were captured under an Olympus BX53 microscope (Evident, Tokyo, Japan) at 400 × magnification. Three random fields per section were analyzed using ImageProPlus software (Media Cybernetics, Rockville, MD, USA). The activation ratio of mast cells was calculated as the proportion of CD63/tryptase double‐positive cells, which serves as an indirect measure of activation [16]. For other targets, mean fluorescence intensity was measured.

### 2.7. Enzyme‐Linked Immunosorbent Assay (ELISA)

Cutaneous specimens were fully homogenized in ice‐cold radioimmunoprecipitation assay (RIPA) lysis buffer (Beyotime, Shanghai, China) at 10 μL/mg tissue weight. After complete lysis, homogenates were centrifuged at 12,000 × g for 5 min at 4°C to pellet debris. The resulting supernatants were collected, and total protein content was quantified with a bicinchoninic acid assay (BCA) kit (Beyotime, Shanghai, China) following the manufacturer’s instructions. All sample concentrations were then adjusted to equal protein levels for subsequent detection. The levels of tumor necrosis factor (TNF)‐α, interleukin (IL)‐6, and IL‐1β were measured using ELISA kits (BOSTER, Wuhan, Hubei, China), while 5‐hydroxytryptamine (5‐HT), CGRP, SP, and histamine (HA) were measured using separate ELISA kits (Meixuan, Shanghai, China), all according to the manufacturers’ protocols. Optical density values at 450 nm were detected via a microplate reader (Thermo Fisher, USA), and the actual concentrations were calculated against corresponding standard curves. Each biological sample was tested in duplicate to guarantee reliable experimental outcomes.

### 2.8. Quantitative Reverse Transcription‐Polymerase Chain Reaction (qRT‐PCR)

Total RNA was extracted from skin and DRG tissues using RNAiso Plus (TaKaRa Bio, Japan). Reverse transcription was performed using a PrimeScript RT reagent kit with gDNA Eraser (TaKaRa Bio, Japan) according to the manufacturer’s instructions. qRT‐PCR was performed using SYBR Premix Ex Taq (TaKaRa Bio, Japan) on a real‐time PCR system (Bio‐Rad, Hercules, CA, USA). The PCR cycling conditions were initiated with a predenaturation step at 95°C for 5 min. Then, this step was followed by 40 cycles of denaturation at 95°C for 15 s and annealing/extension at 60°C for 30 s. The relative expression levels of p38 MAPK, ERK, JNK, Piezo1, and Piezo2 were calculated using the 2^−ΔΔCt^ method, with GAPDH serving as the internal reference gene. Each biological sample was analyzed in technical triplicate. The primers used in the study are listed in Table 1.

**TABLE 1:** Primer sequences used for qRT‐PCR analysis.

Gene name	Forward primer sequence (5′–3′)	Reverse primer sequence (5′–3′)
JNK	CAGCTTATGATGCTATTCTTGAAAGA	AACTAGTTCTCGGTAGGCTCG
p38	GATGCTGGTTTTGGACTCGG	ACTGGTCATAAGGGTCAGCC
ERK	GAGATGCGCTTCACTGTGAG	GCTGACTTCTTGGTGGCAAT
GAPDH	ACTCTACCCACGGCAAGTTC	TGGGTTTCCCGTTGATGACC
Piezo1	ACCCACTAGCCATGCAGTTC	GTATACTCCACAGAGCCGCC
Piezo2	GACAGGCCCTACTTTCCACC	CCGAGGGACAGCTCATCATC

### 2.9. Western Blot

Western blotting was performed to detect the protein expression and phosphorylation levels of ERK, JNK, and p38 in the DRG tissue of each group. Total protein was retrieved from DRG lysates and quantitatively analyzed for equal loading. Samples were separated by sodium dodecyl sulfate‐polyacrylamide gel electrophoresis (SDS‐PAGE) (Beyotime, Shanghai, China) and transferred onto polyvinylidene fluoride membranes. After blocking with 5% BSA in Tris‐buffered saline with Tween 20 (TBST), the membranes were incubated overnight at 4°C with primary antibodies against p38 MAPK (1:1000, 14064‐1‐AP, Proteintech, Wuhan, Hubei, China), p‐p38 MAPK (1:1000, 28796‐1‐AP, Proteintech, Wuhan, Hubei, China), JNK (1:1000, 66210‐1‐Ig, Proteintech, Wuhan, Hubei, China), p‐JNK (1:1000, 80024‐1‐RR, Proteintech, Wuhan, Hubei, China), ERK (1:1000, 11257‐1‐AP, Proteintech, Wuhan, Hubei, China), p‐ERK (1:1000, 28733‐1‐AP, Proteintech, Wuhan, Hubei, China), and GAPDH (1:5000, 5174, Cell Signaling Technology, Danvers, MA, USA). Following three TBST rinses, the membranes were incubated with an HRP‐conjugated secondary antibody (1:5000, Sangon Biotech, Shanghai, China) at room temperature for 2 h. Results were visualized using the enhanced chemiluminescence (ECL) (Beyotime, Shanghai, China) and the ChemiDoc MP system (Bio‐Rad, Hercules, CA, USA). To accurately reflect the absolute changes in intracellular signaling intensity and prevent potential masking effects from total protein fluctuations, the densitometry values of both phosphorylated and total MAPKs were independently normalized to the internal control GAPDH. Densitometry was carried out with ImageJ (Bethesda, MD, USA).

### 2.10. Toluidine Blue Staining

Skin samples from the acupoint area were fixed, routinely dehydrated, and embedded in paraffin. Longitudinal continuous sections were prepared and stained with 0.5% toluidine blue solution. Following dehydration and clearing, sections were mounted with neutral resin. Mast cell degranulation was observed and quantified under an Olympus BX53 microscope (Evident, Tokyo, Japan) at 200 × magnification. Mast cells with intact cell membranes and clear nuclei staining were classified as stable, whereas cells with ruptured membranes and surrounding extruded blue granules were classified as degranulated [17]. Three randomly selected fields of view were examined from each slide to determine the degranulation rate of mast cells under 200 × magnification.

### 2.11. Statistical Analysis

Statistical analysis was performed using SPSS 21.0 software (IBM, Chicago, IL, USA). Normality and homogeneity of variance were first evaluated using the Shapiro–Wilk test and Levene’s test, respectively. Normally distributed data were presented as mean ± standard deviation (SD). For intergroup comparisons at a single time point, one‐way analysis of variance (ANOVA) with least significant difference (LSD) post hoc test was used when variances were homogeneous. When variances were not homogeneous, Dunnett’s T3 test was applied. For the same observational metric across multiple time points, repeated‐measures ANOVA was conducted. When the omnibus test was significant, pairwise intergroup comparisons at each time point were performed using one‐way ANOVA followed by LSD post hoc test. A *p* value < 0.05 was considered statistically significant.

## 3. Results

### 3.1. Mechanical Pain Threshold in CCD Rats

PWT was measured using an electronic von Frey device to evaluate the effect of MT on mechanical allodynia. As shown in Figure 1, baseline PWT values were similar across all groups. From day 3 after CCD surgery, the PWT in the CCD group was markedly lower than that in the sham group (*p* < 0.01). Intervention was initiated on day 4 in the MT group. From day 11 onward, the PWT in the MT group was significantly higher than that in the CCD group (*p* < 0.01) (Figure 1), and the PWT continued to rise until day 18. In the MCI group, acupoint injection of cromolyn sodium began on day 4. From day 11 to day 18, the PWT in the MCI group was also significantly higher than that in the CCD group (*p* < 0.01) (Figure 1) but gradually declined from day 14 to day 18. On day 18, although the PWT in the MCI group remained significantly higher than that in the CCD group (*p* < 0.01), it was significantly lower than that in the MT group (*p* < 0.05) (Figure 1). These data indicate that both MT and MCI substantially elevate the mechanical pain threshold in CCD rats, but the analgesic effect of MT is more sustained than that achieved by mast cell inhibition alone.

**FIGURE 1:**
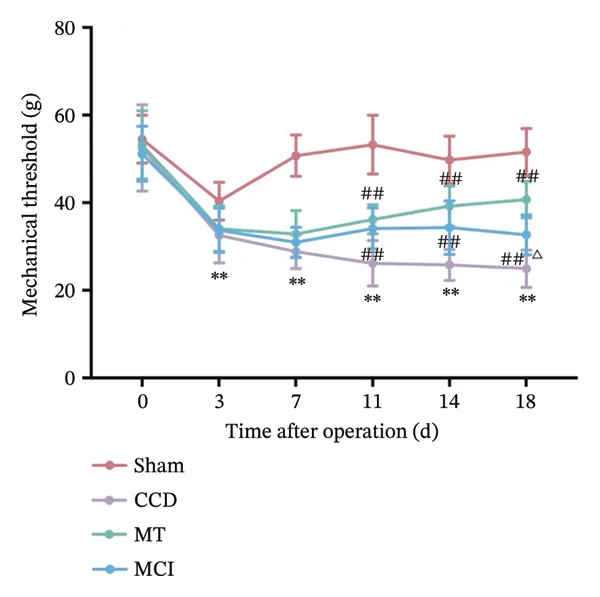
Comparison of mechanical pain thresholds among different groups of rats, *n* = 6 rats/group. ^∗∗^ *p* < 0.01 vs. sham at days 3, 7, 11, 14, and 18; ^##^ *p* < 0.01 vs. CCD at days 11, 14, and 18; ^Δ^ *p* < 0.05 vs. MT at day 18 only. Data are presented as mean ± SD; error bars represent SD. Repeated‐measures ANOVA with LSD post hoc test was used. Abbreviations: CCD: chronic compression of dorsal root ganglion, MT: manual therapy, MCI: mast cell inhibition.

### 3.2. Mast Cell Activation in the Skin

Immunofluorescence was performed to examine the activation status of mast cells in the skin. Tryptase was used to label mast cells, and CD63 was used to determine their activation state, serving as an indirect measure of activation. As shown in Figure 2, compared with the sham group, the proportion of activated mast cells (CD63/Tryptase) in the CCD group was significantly increased (*p* < 0.01). Following MT and MCI intervention, the proportion of activated mast cells in the skin was markedly reduced compared with the CCD group (*p* < 0.01) (Figure 2a,d).

**FIGURE 2:**
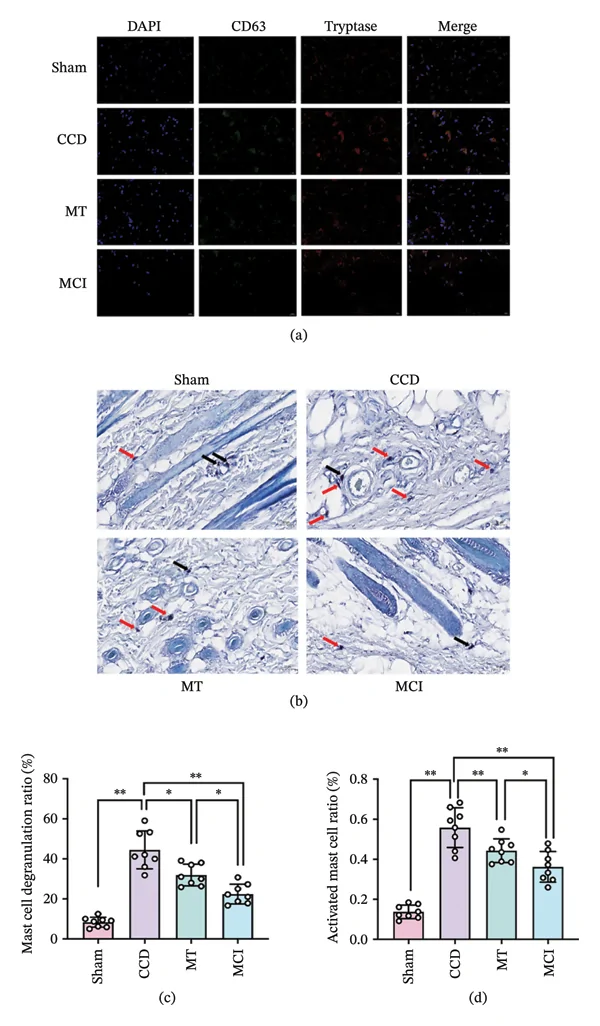
Comparison of activated mast cells in the skin among groups. (a) Immunofluorescence images (400x). Scale bar = 20 µm; (b) representative images of toluidine blue staining in cutaneous tissues (200x). Black arrows indicate stable mast cells; red arrows indicate degranulated mast cells. Scale bar = 50 µm; (c) proportion of mast cell degranulation, *n* = 8 rats/group; (d) proportion of activated mast cells, *n* = 8 rats/group; data are presented as mean ± SD. Error bars represent the SD. ^∗^ *p* < 0.05, ^∗∗^ *p* < 0.01. Abbreviations: CCD: chronic compression of dorsal root ganglion, MT: manual therapy, MCI: mast cell inhibition.

Toluidine blue staining was performed to evaluate cutaneous mast cells degranulation to provide morphological evidence of mast cell activation. In the sham group, mast cells were sparse, structurally intact, and rarely exhibited degranulation. Compared with the sham group, mast cells in the CCD group exhibited disrupted morphology, such as loose structures and extruded granules, resulting in a markedly higher degranulation ratio (*p* < 0.01). Both MT and MCI interventions attenuated these pathological changes. MT yielded more intact mast cell morphology and a significantly reduced degranulation ratio compared to CCD group (*p* < 0.05). MCI intervention further restricted mast cell degranulation, demonstrating a statistically superior inhibitory effect compared to MT (*p* < 0.05) (Figure 2b,c). Together, these results suggest that both MT and MCI are associated with reduced proportion of activated mast cells in the skin.

### 3.3. Levels of Inflammatory Cytokines in the Skin

ELISA was used to detect the levels of inflammatory cytokines in the skin. As shown in Figure 3, compared with the sham group, the levels of IL‐1β, IL‐6, and TNF‐α in the CCD group were significantly increased (*p* < 0.01). Both MT and MCI significantly reduced the expression of these cytokines compared with the CCD group (*p* < 0.01) (Figure 3). These results indicate that MT and MCI significantly attenuate CCD‐induced upregulation of pro‐inflammatory cytokines in the skin.

**FIGURE 3:**
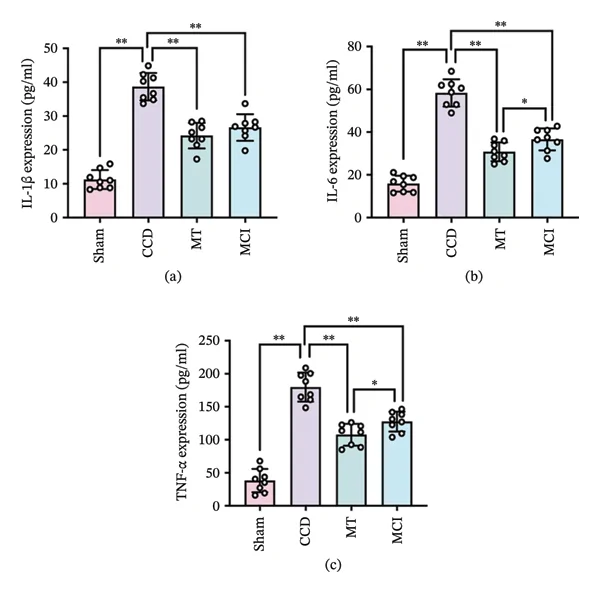
Changes of inflammatory cytokines in the skin in each group of rats. (a) IL‐1β expression level; (b) IL‐6 expression level; (c) TNF‐α expression level, *n* = 8 rats/group; data are presented as mean ± SD. Error bars represent the SD. ^∗^ *p* < 0.05, ^∗∗^ *p* < 0.01. Abbreviations: CCD: chronic compression of dorsal root ganglion, IL‐1β: interleukin‐1 beta, IL‐6: interleukin‐6, MT: manual therapy, MCI: mast cell inhibition, TNF‐α: tumor necrosis factor‐alpha.

### 3.4. Levels of Neurotransmitters in the Skin

ELISA was used to detect the levels of neurotransmitters, while immunofluorescence was performed to evaluate the protein expression of CGRP and SP in the skin. As shown in Figure 4, compared with the sham group, the levels of neurotransmitters (CGRP, SP, 5‐HT, and HA) in the CCD group were significantly increased (*p* < 0.01). Compared with the CCD group, both the MT group and MCI group showed significantly reduced expression of neurotransmitters in the skin (*p* < 0.01). Consistently, immunofluorescence revealed enhanced CGRP and SP immunoreactivity in the skin of CCD rats, which was markedly attenuated by MT and MCI intervention (Figure 4a). These results suggest that MT and MCI significantly attenuate CCD‐induced upregulation of neurotransmitters in the skin.

**FIGURE 4:**
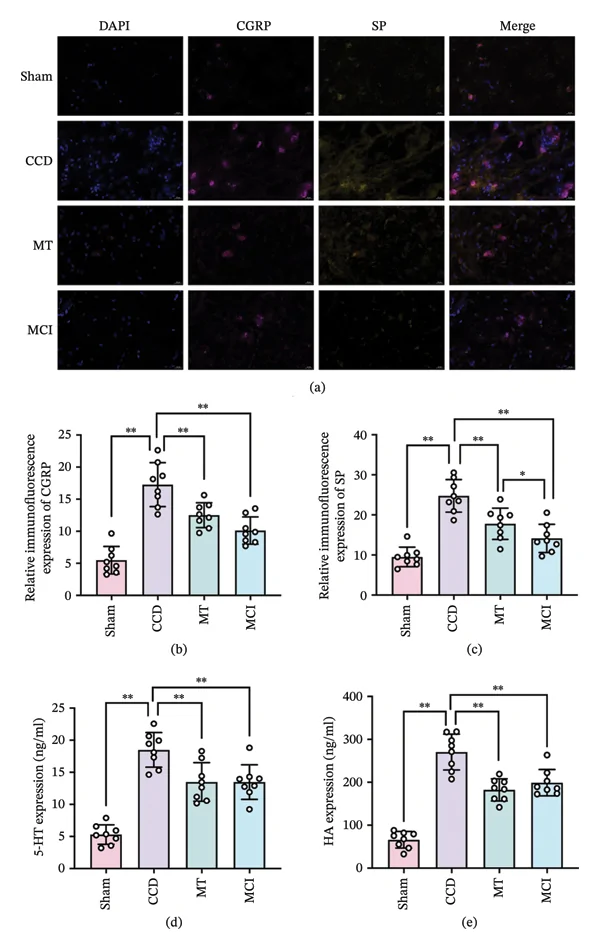
Comparison of neurotransmitters in the skin in each group of rats. (a) Immunofluorescence images (400x). Scale bar = 20 µm; (b) relative immunofluorescence expression of CGRP; (c) relative immunofluorescence expression of SP; (d) 5‐HT expression level; (e) HA expression level, *n* = 8 rats/group; data are presented as mean ± SD. Error bars represent the SD. ^∗^ *p* < 0.05, ^∗∗^ *p* < 0.01. Abbreviations: CCD: chronic compression of dorsal root ganglion, 5‐HT: 5‐hydroxytryptamine, HA: histamine, MT: manual therapy, MCI: mast cell inhibition, SP: substance P, CGRP: calcitonin gene‐related peptide.

### 3.5. Phosphorylation of MAPK Pathways in the DRG

Western blotting was used to detect the total and phosphorylation protein levels of p38 MAPK, ERK, and JNK in the DRG tissue of each group, while qRT‐PCR was performed to measure their mRNA expression. As shown in Figure 5d–f,j–l, no significant differences were observed in the total protein and mRNA expression levels of p38 MAPK, ERK, or JNK among the groups. However, compared with the sham group, the levels of p‐p38 MAPK, p‐ERK, and p‐JNK in the CCD group were significantly increased (*p* < 0.01). Both the MT and MCI groups significantly reduced the expression levels of p‐p38 MAPK, p‐ERK, and p‐JNK in the DRG compared with the CCD group (*p* < 0.01) (Figure 5g–i). These results indicate that both MT and MCI significantly reduce the phosphorylation of p38 MAPK, ERK, and JNK in the MAPK pathway.

**FIGURE 5:**
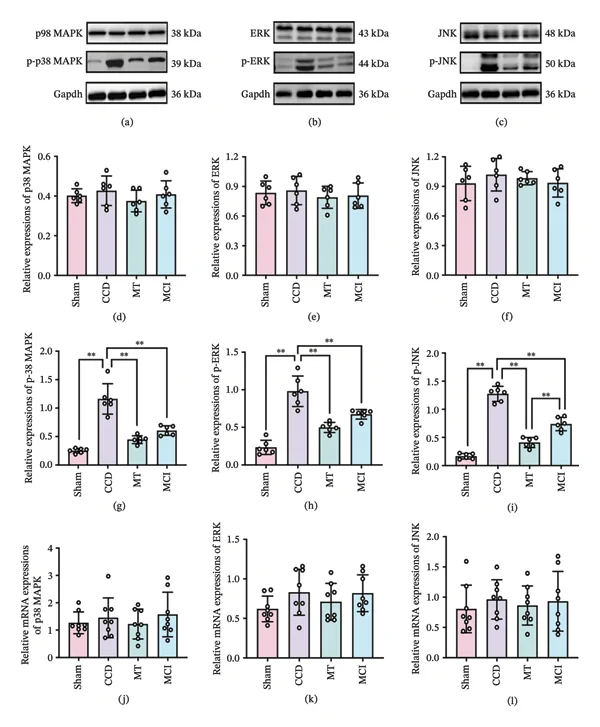
Expression and phosphorylation of ERK, JNK, and p38 in DRG tissue of rats in each group. (a–c) Representative western blot bands; (d) relative expression levels of p38 MAPK; (e) relative expression levels of ERK; (f) relative expression levels of JNK; (g) relative expression levels of p‐p38 MAPK; (h) relative expression levels of p‐ERK; (i) relative expression levels of p‐JNK. *n* = 6 rats/group; (j) relative mRNA expression levels of p38 MAPK; (k) relative mRNA expression levels of ERK; (l) relative mRNA expression levels of JNK, *n* = 8 rats/group; data are presented as mean ± SD. Error bars represent the SD. ^∗∗^ *p* < 0.01. Abbreviations: CCD: chronic compression of dorsal root ganglion, MT: manual therapy, MCI: mast cell inhibition.

### 3.6. Expression of Piezo1 and Piezo2 in the Skin and DRG

Immunofluorescence and qRT‐PCR were performed to detect the protein and mRNA expression levels of Piezo1 and Piezo2 in the skin and DRG. As shown in Figure 6b,d, for Piezo1, no significant differences in relative fluorescence intensity and mRNA expression were observed in the skin among all four groups. As shown in Figure 6g,i, compared with the sham group, the relative fluorescence intensity and mRNA expression in the DRG of CCD, MT, and MCI groups were all decreased. The CCD group showed a significant difference from the sham group (*p* < 0.01). For Piezo2, in both tissues, the relative fluorescence intensity and mRNA expression in the CCD, MT, and MCI groups were all increased compared with the sham group. The CCD group showed significantly higher expression than the sham group (*p* < 0.01), whereas MT group exhibited significantly lower expression than the CCD group (*p* < 0.01) and the MCI group (Figure 6c,e,h,j). These results indicate that Piezo2 downregulation is associated with the analgesic effects of both MT and MCI. However, the sustained behavioral superiority of MT over MCI suggests that additional mechanisms beyond Piezo2 modulation may also be involved.

FIGURE 6Analysis of Piezo1 and Piezo2 expression in the skin and DRG among groups. (a) Immunofluorescence images in the skin (400×). Scale bar = 20 µm; (b) Piezo1 immunofluorescence expression in the skin; (c) Piezo2 immunofluorescence expression in the skin, *n* = 8 rats/group; (d) Piezo1 mRNA expression in the skin; (e) Piezo2 mRNA expression in the skin, *n* = 6 rats/group; (f) Immunofluorescence images in the DRG (400×). Scale bar = 20 µm; (g) Piezo1 immunofluorescence expression in the DRG; (h) Piezo2 immunofluorescence expression in the DRG, *n* = 8 rats/group; (i) Piezo1 mRNA expression in the DRG; (j) Piezo2 mRNA expression in the DRG, *n* = 6 rats/group; data are presented as mean ± SD. Error bars represent the SD. ^∗^ *p* < 0.05, ^∗∗^ *p* < 0.01. Abbreviations: CCD: chronic compression of dorsal root ganglion, MT: manual therapy, MCI: mast cell inhibition.

**Figure:**
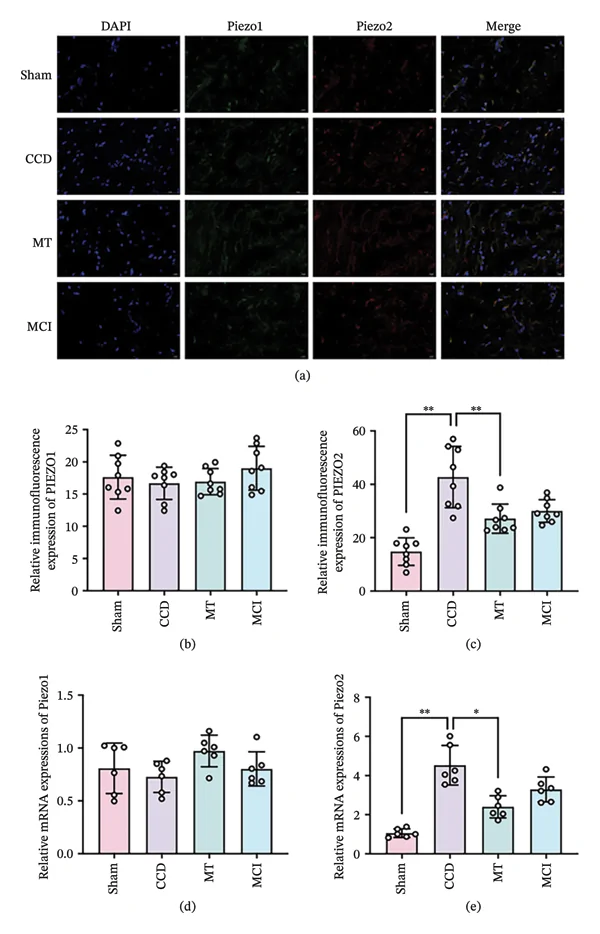

**Figure:**
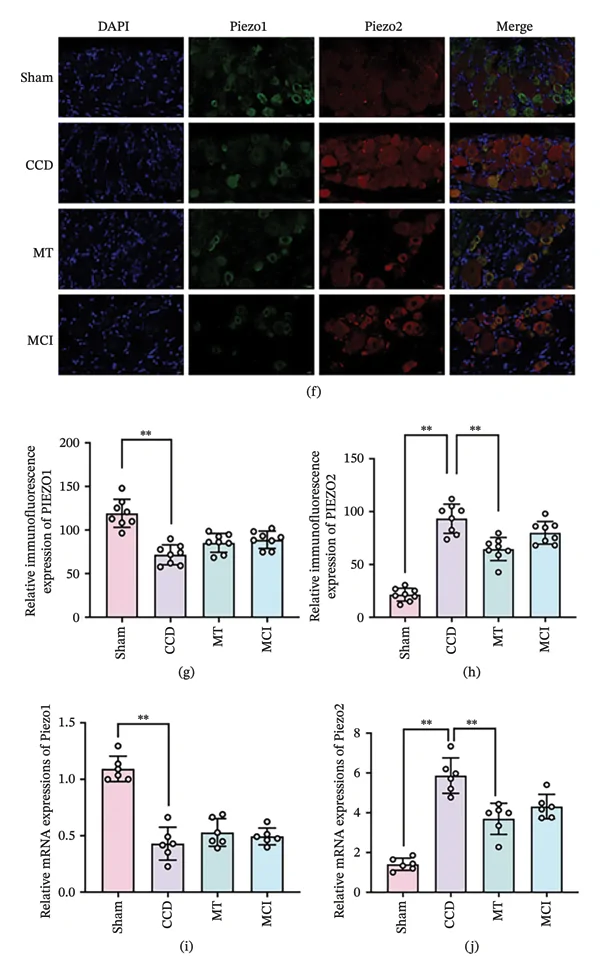

## 4. Discussion

The present study explored the analgesic efficacy and underlying molecular mechanism of MT against CCD‐induced mechanical allodynia, using the MCI to examine whether MT acts through mast cell modulation. Consistent with behavioral observations, the biological analyses from peripheral skin to DRG indicated that MT may alleviate CCD‐triggered neuropathic pain. These analgesic effects were associated with suppressed mast cell overactivation, attenuated peripheral inflammatory cascades, reduced MAPK phosphorylation, and decreased Piezo2 expression. These findings provide novel evidence that cutaneous mast cells may act as key peripheral responders to mechanical stimulation, serving as a biological bridge between MT and local neuro‐immune modulation.

The low back and leg pain caused by LDH primarily stems from pathological changes such as mechanical compression of nerve roots by the herniated nucleus pulposus and subsequent inflammatory reactions. MT is a widely used conservative therapy for LDH in clinical practice [18, 19]. According to TCM theory, stimulation of specific points on the body surface can exert regulatory effects on internal organs and treat distant disorders [19, 20]. This theory posits a bidirectional communication between superficial body areas and internal organs, with acupoints serving as both reaction points for visceral diseases and portals for therapeutic intervention [20]. Although current studies focusing on the local skin response to MT are still limited, existing research suggests that inflammatory cytokine levels are elevated at cutaneous reaction points in LDH patients [5], which may be closely associated with pain. The BL57, an important pain reaction point in the lower limb, is anatomically associated with the L4‐S1 spinal segments commonly involved in LDH and MT at BL57 has been shown to produce significant clinical analgesic effects [21]. Our previous clinical work [7, 8] also demonstrated that MT at BL57 effectively relieves pain in LDH patients. Therefore, this research selected the skin over the BL57 acupoint as the intervention target to more intuitively observe local tissue responses and to explore the mechanism by which MT exerts local analgesic effects.

The CCD model reliably reproduces the neuropathic pain state following sustained nerve root compression, as demonstrated by our previous studies [22, 23]. Behavioral results showed that PWT in the CCD group markedly decreased from day 3 and stabilized by day 14. From day 11 onward, PWT in the MT group was significantly higher than that in the CCD group, indicating that MT effectively improves mechanical allodynia in CCD rats. To further verify the role of mast cells, an MCI group was included. In this group, PWT also became significantly higher than that in the CCD group from day 11 but remained significantly lower than that in the MT group on day 18. These results indicate that MT may alleviate pain by suppressing mast cell activation.

A previous study [24] has indicated that the formation of body surface reaction points in response to internal diseases is closely related to local neurogenic inflammation. Among the cellular components involved in this process, mast cells are considered key mediators of neural signaling and immune‐inflammatory responses due to their anatomical proximity to sensory nerve endings and high‐density distribution in acupoint regions [25–27]. In line with this, pharmacological stabilization in other models [28–30] and nonpharmacological therapies, including acupuncture [31], are associated with analgesic and anti‐neuroinflammatory outcomes alongside reduced mast cell degranulation. However, whether MT, as a mechanical stimulus, exerts analgesic effects through the same mechanism remains underexplored. To address this gap, this study measured specific markers of mast cells and their degranulation. Toluidine blue staining demonstrated extensive mast cell degranulation following CCD, which was attenuated by both MT and MCI. Tryptase specifically identifies mast cells, while CD63 translocates to the surface upon activation. While reliable, this dual labeling indirectly assesses activation. It cannot directly visualize granule exocytosis or fully quantify mediator release. Immunofluorescence showed elevated CD63/tryptase co‐localization in CCD rats, which was also significantly attenuated by both MT and MCI. The histological observations align with the immunofluorescence data, collectively suggesting that MT may help limit mast cell activation.

The research further investigated the upstream signals and downstream molecular networks. Chronically compressed neurons release neuropeptides such as SP and CGRP via axonal retrograde transport to the innervated acupoint region, where they act on mast cell receptors. This induces degranulation and a local inflammatory cascade [9, 32]. Consistent with this mechanism, our experiments demonstrated that SP and CGRP were significantly elevated in the skin of CCD rats. Both MT and MCI effectively downregulated them. Since mast cell–derived mediators such as 5‐HT can feed back onto sensory nerve endings to promote further SP and CGRP release [33], the inhibition of mast cell activation by MT would break this positive feedback loop, thereby reducing neuropeptide overflow. This suggests that MT may suppress excessive neuropeptide release by inhibiting mast cell activation. The disruption of this neuro‐immune feedback loop attenuates pain signal transmission and acupoint sensitization, ultimately reversing the pro‐nociceptive local microenvironment [34–36]. This pathological feedback loop explains the increased inflammatory cytokine levels in the skin of the CCD group. Both interventions decreased downstream inflammatory mediators and neurotransmitters. Thus, MT may attenuate peripheral sensitization by modulating mast cell activation and disrupting the neuro‐immune regulatory circuit.

The inflammatory mediators released from activated mast cells act both locally and remotely, influencing the DRG, where they drive intracellular signaling cascades that promote neuronal sensitization. Among these cascades, the MAPK signaling cascade in the DRG is established as a central intracellular pathway governing nociceptive signal transduction following peripheral nerve injury [37]. Upon activation, phosphorylated MAPKs translocate into the nucleus and initiate transcription of pain‐associated genes, a process that exacerbates peripheral sensitization in DRG. In this study, CCD rats showed markedly increased phosphorylation of MAPK members (ERK, JNK, p38 MAPK) in the DRG, indicating sustained activation of this pro‐nociceptive signaling. Both MT and MCI significantly reduced p‐ERK, p‐JNK, and p‐p38 MAPK levels. Given that MAPK activation promotes transcription of inflammatory cytokines such as TNF‐α, IL‐1β, and IL‐6 [38, 39], the suppression of MAPK phosphorylation likely contributes to reduced inflammatory cytokine levels in DRG neurons. This may dampen nociceptive transmission to the spinal cord, thereby alleviating pain. Collectively, these results suggest that MT is associated with attenuated DRG MAPK hyperactivation, which occurs alongside the restriction of cutaneous mast cell–driven peripheral inflammatory input.

Additionally, mechanosensitive ion channels have gained attention as potential mediators of MT. Given that MT is a mechanical stimulus, this study explored the expression of Piezo1 and Piezo2, two mechanically gated channels implicated in mechanotransduction. Piezo2 mediates light touch and has been linked to mechanical allodynia under neuropathic pain conditions, converting mechanical forces into nociceptive signals [40], whereas Piezo1 is primarily involved in vascular and non‐neuronal mechanotransduction [41]. Consistent with their distinct functions, Piezo2 is predominantly expressed in nociceptive sensory neurons and their peripheral endings, while Piezo1 is highly expressed in vascular endothelial cells and erythrocytes, with limited presence in pain‐signaling neurons [42]. Our results experimentally demonstrated that both MT and MCI attenuate CCD‐associated Piezo2 upregulation in both tissues, while Piezo1 expression remained unchanged in the skin. This suggests that Piezo2 downregulation may contribute to the observed analgesic effects. However, the precise causal sequence linking mechanical stimulation, Piezo2, and mast cells remains hypothetical. Given that MCI alone did not fully replicate the sustained behavioral effect of MT, the differential regulation of Piezo2 by MT may contribute to this discrepancy. MT may engage additional pathways beyond mast cell inhibition, potentially via effects on sensory neuron mechanotransduction or other mechanosensitive targets. As we did not employ selective Piezo2 agonists or antagonists to establish causality, these findings remain correlative and should be interpreted with caution. Future studies using pharmacological or genetic tools to manipulate Piezo2 activity are required to determine whether and how Piezo2 contributes to the sustained analgesic effect of MT. In addition, the downregulation of Piezo1 in the DRG may reflect a compensatory response to chronic compression, though its functional implications remain unclear and warrant further investigation.

Nonetheless, several limitations of this study must be acknowledged. The observed link between cutaneous mast cells and DRG MAPK changes remains strictly associative. The present study focused on peripheral tissues and did not examine indicators of neuroinflammation and pain modulation in the central nervous system, such as the spinal dorsal horn. While the DRG findings suggest that MT attenuates peripheral nociceptive signaling, this study did not assess dorsal horn neuronal excitability or glial activation. Therefore, how the local peripheral effects further influence central sensitization remains to be explored. In addition, our findings on Piezo1 and Piezo2 remain speculative. Without genetic knockouts or selective antagonists, their exact causal role cannot be confirmed. The interplay between these mechanosensitive channels and mast cell activation requires dedicated investigation. Future studies combining specific Piezo agonists or antagonists with mast cell modulators are needed to clarify their respective roles. Additionally, relying on a single inhibitor introduces potential off‐target effects. Using mast cell–deficient models would better isolate their specific role. Finally, the CCD model used in this study predominantly mimics nerve root compression, whereas the clinical pathology of LDH is more complex and multifactorial. Hence, future studies could incorporate additional animal models that better simulate the multifactorial pathology of LDH and include sex‐specific control groups to address potential sex differences in pain. This may elucidate the molecular signaling pathways and neural circuits involved in MT analgesia and promote the clinical translation of these findings.

## 5. Conclusions

In summary, this study demonstrates that cutaneous mast cells contribute to the analgesic effect of MT on CCD rats. MT may suppress mast cell activation and attenuate the local inflammatory microenvironment, which correlates with reduced MAPK phosphorylation in the DRG and diminished peripheral sensitization (Figure 7). Collectively, these findings identify cutaneous mast cells as responders to mechanical stimulation and their inhibition may contribute to the analgesic effect of MT. This points to future directions for investigating the molecular mechanisms of MT’s multitarget regulation.

**FIGURE 7:**
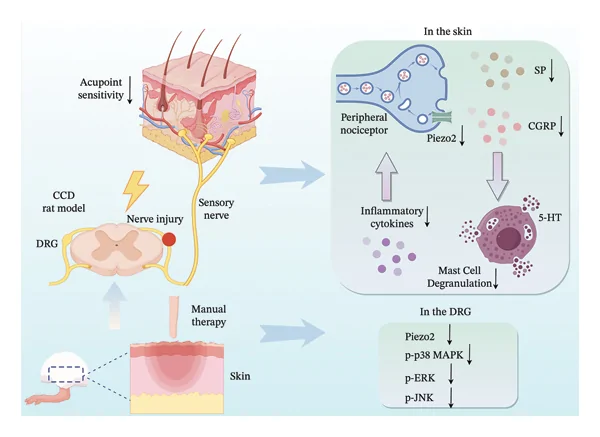
Cutaneous mast cells contribute to the analgesic effect of manual therapy on CCD rats. Abbreviations: CCD: chronic compression of dorsal root ganglion, 5‐HT: 5‐hydroxytryptamine, DRG: dorsal root ganglion, SP: substance P, CGRP: calcitonin gene‐related peptide.

## Author Contributions

Chongjie Yao and Jiayang Yu designed and conducted the animal experiments. Chongjie Yao and Dong Xie conducted indicator measurement and statistical analysis. Jiayang Yu and Pingping Sun drafted and revised the manuscript. Xinyu Zeng, Xili Chang, and Wuquan Sun performed the data collection and analysis. Min Fang, Qingguang Zhu, and Chongjie Yao were responsible for guiding and reviewing this article.

## Funding

This work was supported by the National Natural Science Foundation of China (82305423, 82575242, 82575245, 82474671), Construction Project of High level Key Traditional Chinese Medicine Disciplines of the National Administration of Traditional Chinese Medicine (zyyzdxk‐2023061), Yangfan Special Project of Shanghai Science and Technology Innovation Action Plan (24YF2740700), and Shanghai Key Laboratory of Traditional Chinese Medicine Tuina Techniques for Musculoskeletal Disorders (24dz2260200).

## Conflicts of Interest

The authors declare no conflicts of interest.

## Data Availability

The data that support the findings of this study are available from the corresponding author upon reasonable request.
